# Health and social needs of older adults in slum communities in Ghana: a phenomenological approach used in 2021

**DOI:** 10.1186/s13690-023-01056-9

**Published:** 2023-04-27

**Authors:** Priscilla Yeye Adumoah Attafuah, Irma HJ Everink, Christa Lohrmann, Aaron Abuosi, Jos MGA Schols

**Affiliations:** 1 School of Nursing and Midwifery, University of Ghana, Legon, Ghana; 2 Department of Health Services Research and Care and Public Health Research Institute (CAPHRI), Maastricht University, Maastricht, the Netherlands; 3 Institute of Nursing Science, Medical University of Graz, Graz, Austria; 4 Health Services Management Department, University of Ghana Business School, Legon, Ghana; 5 Department of Family Medicine and Care and Public Health Research Institute (CAPHRI), Maastricht University, Maastricht, the Netherlands

**Keywords:** Health needs, Old age and Social Care, Social needs, Social and Health Services, Quality-of-life, Urban slums, Ghana

## Abstract

Slum-dwellers lack several essential amenities (such as water, sanitation, and electricity) which make them more vulnerable than non-slum dwellers. As there is limited to no access to health and social care services in slums, the slum environment is expected to be an even more dangerous environment for older adults, negatively impacting their quality of life (QoL). To provide an overview of the perceived (unmet) health and social care needs and how it affects the QoL, this study aims to explore the self-perceived health and social needs of older adults in urban slums in Ghana. Using a phenomenological approach, 25 semi-structured interviews were conducted between May and June 2021, in the homes of older adults in two slums in Ghana. After coding and analysing the transcripts, five main themes emerged: (a) perception of health; (b) (de)motivators of health service use; (c) perception of social care, (d) social needs, and (e) influence of phenomena on QoL. It appeared that older adults believed that spiritual powers were causing illnesses and influenced their use of formal health services. Other factors such as expired insurance cards and the attitude of healthcare workers served as demotivators for using health services.

Perceived health needs were mainly current disease conditions (arthritis, diabetes, hypertension, vision/hearing challenges), challenges with health insurance, the behaviour of some health professionals, the proximity of health facilities, and unnecessary queues at major health facilities. Unmet social needs identified by this study were a sense of neglect by family (need for companionship), requiring assistance with activities of daily living, and the need for financial support. Participants had more health needs than social needs. Health providers do not usually prioritize the care of slum-dwelling older adults. Most participants still have challenges with the National Health Insurance Scheme (NHIS). Their social needs were mainly related to financial difficulties and help with some activities of daily living. Participants expressed that they desired companionship (especially the widowed or divorced ones) and the lack of it made them feel lonely and neglected. Home visits by health professionals to older adults should be encouraged to monitor their health condition and advocate for family members to keep older adults company. Healthcare providers should exhibit positive attitudes and educate older patients on the advantages of formal health services use, as well as the need to seek early treatment as this will influence their QoL to a large extent.

**Supplementary Information:**

The online version contains supplementary material available at 10.1186/s13690-023-01056-9.

## What is known about the topic

- Health, participation, and security can ensure a positive QoL among older adults.
- Slum-dwellers lack many amenities which makes them vulnerable.
- The health and social needs of older adults are numerous.

## What this paper adds

- The identified health and social needs from the view of the slum-dwelling older adult.
- Older adults place much more importance on perceived health-related needs than social needs.
- The social needs of older adults in slums in Ghana are few because the family system still exists. Yet the issue of mobility hinders the participation of slum-dwelling older adults in family activities. This creates a feeling of loneliness and neglect.
- Health education through religious bodies can help provide insight into some health needs of older adults.

## Introduction

Older adults living in developing countries, face various challenges regarding their health and social needs. These challenges are even more profound among older adults living in slum areas in developing countries, because of poor living environments [1–4] and humans are influenced by the environment. Rural-urban migration has resulted in the emergence of slums in the large cities of most developing countries like Ghana. When comparing formal settlements with slums, people living in slums lack basic amenities like water, electricity and proper collection and disposal of solid waste. They are also exposed to health risks by noise pollution, poor sanitation, and hygiene, face poor housing conditions, overcrowding and violence [4–6] and have limited access to health and social care services. The pollution and environmental hazards as well as the uneven road networks in the slums have negative effects on the older adult. As people age, they increasingly need support in various domains, such as mobility, self-care, social participation, and healthcare [7, 8]. As the access to health and social care services, including primary care, disease prevention, rehabilitation and health promotion in slums is limited or non-existent, the basic needs of these older adults are often unmet. Indeed, many parts of urban Ghana can also fall into the criteria for the classification of slums because of the level of resources available in Ghana as well as poor planning for putting up structures in most parts of Ghana. For this reason, certain places have more slum characteristics than others. The slum concept for this study is based on the UN-Habitat classification of slums [5]. This states that any household with one or more of the following is classified as a slum: (1) Lack of access to an improved water source; (2) Lack of access to improved sanitation facilities; (3) Lack of sufficient living area; (4) Lack of housing durability; and (5) Lack of security of tenure. This could highly influence the quality of life (QoL) of these slum-dwellers as they age [2].

Quality of life (QoL) according to the World Health Organisation Quality of Life Group, [9] is “an individual’s perception of their position in life in the context of the culture and value systems in which they live and in relation to their goals, expectations, standards and concerns”. Therefore, in the context of living in slums, this study focused on older adults to explore their perception of their health and social needs as a necessary component of their entire QoL. Studies have suggested that active ageing is a major contributor to the QoL of individuals [10, 11].

**Figure 1:**
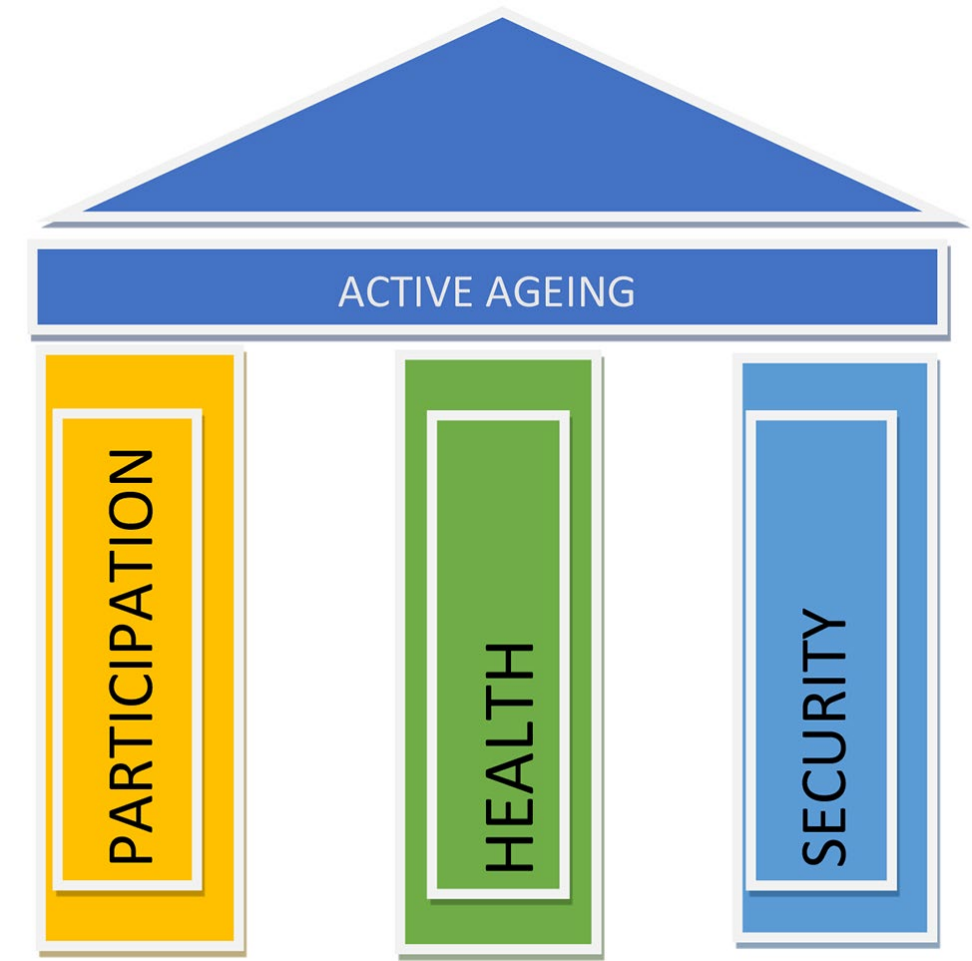
WHO policy framework on Active Ageing

Active ageing is defined by the World Health Organization (WHO) as “the process of optimizing opportunities for health, participation and security in order to enhance the quality of life as people age” [12]. In the concept of active ageing, “active” is not limited to physical activity but the ability to continue being involved in the social, cultural, civic, spiritual, and economic affairs of society. The WHO policy framework on Active Ageing (Figure 1) outlines three pillars that can ensure a positive QoL among older adults [12]: health, participation, and security. The first two pillars were used in the conceptualization of this study. The first pillar, health, refers to an individual’s physical, mental, and social well-being and includes access to healthcare services, nutritional needs and a healthy environment. The second pillar, participation, refers to an individual’s involvement in spiritual, social, cultural, and community affairs. The third pillar, security is out of the scope of this study as it cannot be influenced by healthcare professionals.

Health as defined by the WHO (1948) is the “state of complete physical, mental and social well-being and not merely the absence of disease or infirmity” [13]. Health needs are associated with the treatment, management or prevention of an injury or disability, disease, illness, and the care of an individual. However, every individual can have his/her perception of what being healthy or unhealthy means. When looking at the first pillar, in this population, health issues can be enormous [1, 14–16]. Studies have stated that frequent health problems of older slum-dwellers include depression, physical injuries, malnutrition, chronic diseases, and substance abuse [17–19]. Furthermore, a study in India revealed that social distancing protocols developed during the COVID-19 pandemic were badly implemented in slums, causing higher COVID-19 rates in slums compared to formal settlements [20] mainly because of overcrowding.

Even though these older adults suffer from severe health issues, they seem to make limited use of healthcare services [21, 22]. Their perception of health could be a likely influence on healthcare patronage [14, 18, 57, 63]. Additionally, studies show that the poor financial status of slum dwellers, in combination with the lack of healthcare facilities in the proximity could account for this [23–25]. Uneven walkways and decreased mobility of older adults, also limit access to healthcare services [21, 26, 27]. Still, even though health has a large influence on QoL, little research has been done on the health needs of this group of older slum-dwellers.

When looking at the second pillar, participation, the rationale for this pillar is that social connections are important in the QoL of older adults. The WHO advised to aim at the autonomy and independence of older adults whiles also highlighting that there may be frail, disabled older adults who may need care. In line with this, it appears that the social needs of older people are diverse [27, 28]. Social isolation and loneliness among slum-dwelling older adults can result in a reduction in both mental and physical well-being. Social needs include love, acceptance, and relationships with family and friends. In satisfying these social needs, it is necessary to identify what is classified as a social need among older slum-dwellers. This is because mutuality is important to meet this need. For example, the older adult wants to feel a sense of belonging and connectedness to a community or neighbourhood. Staying active by joining family events or participating in social activities is particularly cherished in countries in Africa [27, 28] and positively impacts their quality of life [27, 29]. In slums in Ghana, recreational centres are rare. Therefore, there are not many options for older slum dwellers to engage in social activities. Also, if older slum-dwellers need social support they mostly rely on family members. However, these may not always be available [29, 30]. Unmet social needs may lead to loneliness and social isolation, which may, in turn, cause psychological and physical health problems [29, 31]. These two concepts: health and (social) participation are the motivators of this phenomenological study among older adults in the slums.

When looking at what is known about (unmet) health needs and social needs, studies mostly focus on populations with specific conditions such as mental illnesses, joint pains, hypertension, and diabetes [32–34]. Additionally, studies focus on different settings, such as rural areas [35], and mostly on populations living in developed countries [36]. However, in sub-Saharan Africa, specifically Ghana, the health, and social needs of older adults in slums have been rarely explored. The uniqueness of this study is that it is from the perspective of older adults living in urban slums. considering the needs and resources available to older adults in rural areas, their counterparts in urban slums may perceive things differently. For example, health facilities may be available but the finances to patronize them is an issue. One study published by Attafuah et al. [37] showed that older adults in slums had a moderate psychological, social, and environmental QoL and a poor physical QoL. Previous studies by the authors on the quality of life of older adults in slums revealed that the health and social needs of this population have not been explored in Ghana. To improve the QoL of this population, it is essential to have, more in-depth information on their (unmet) needs in these domains. Therefore, this study aims to describe the health and social care needs of older adults living in slums and explore how they influence their quality of life.

## Materials and methods

### Study design

A qualitative exploratory descriptive design was used specifically, Husserl’s transcendental phenomenology. This design was chosen as qualitative studies allowed for in-depth exploration of the experiences of the population under consideration [38] and this phenomenological approach constantly assesses the influence of the researcher on the inquiry so that biases and preconceptions are neutralized. Leaning on the constructivism theory as we sought to understand the subjective reality of what health and social care are and their influence on the quality of life from the view of older adults in slums.

### Study setting

**Figure 2:**
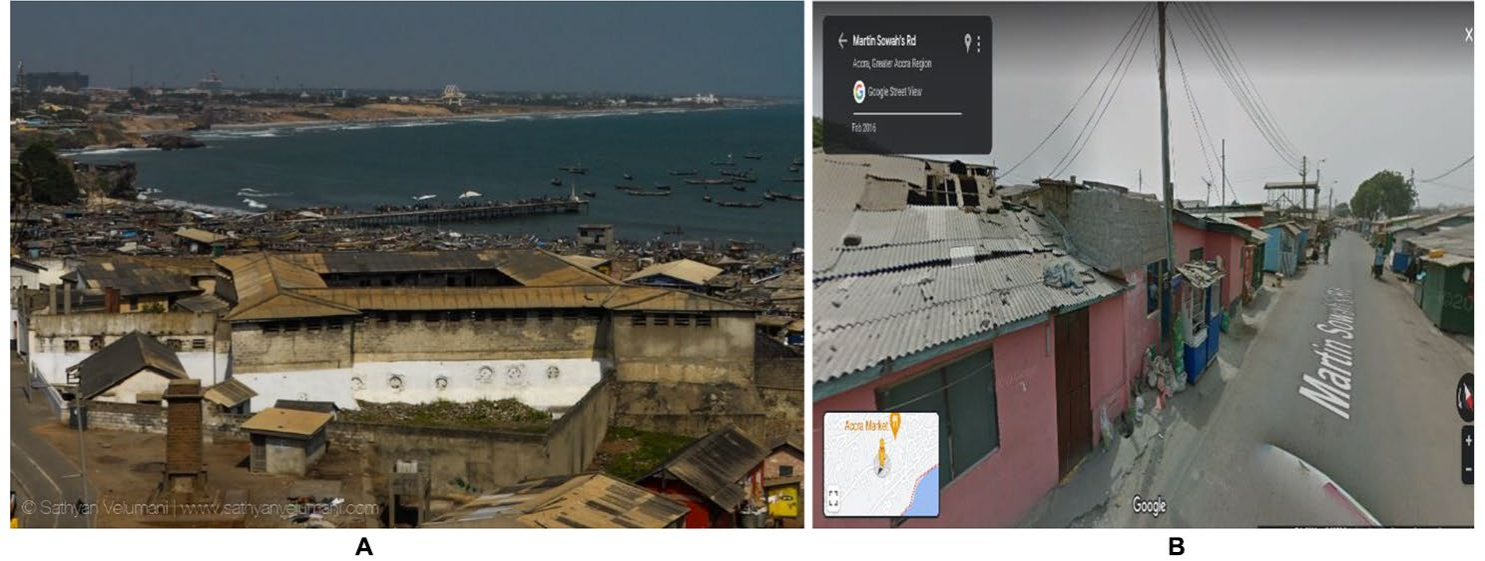
Aerial (A) and street (B) view of Teshie Maami, Ghana (a slum in the Teshie community). Taken in February 2016 and *culled from* Google images in February 2023

**Figure 3:**
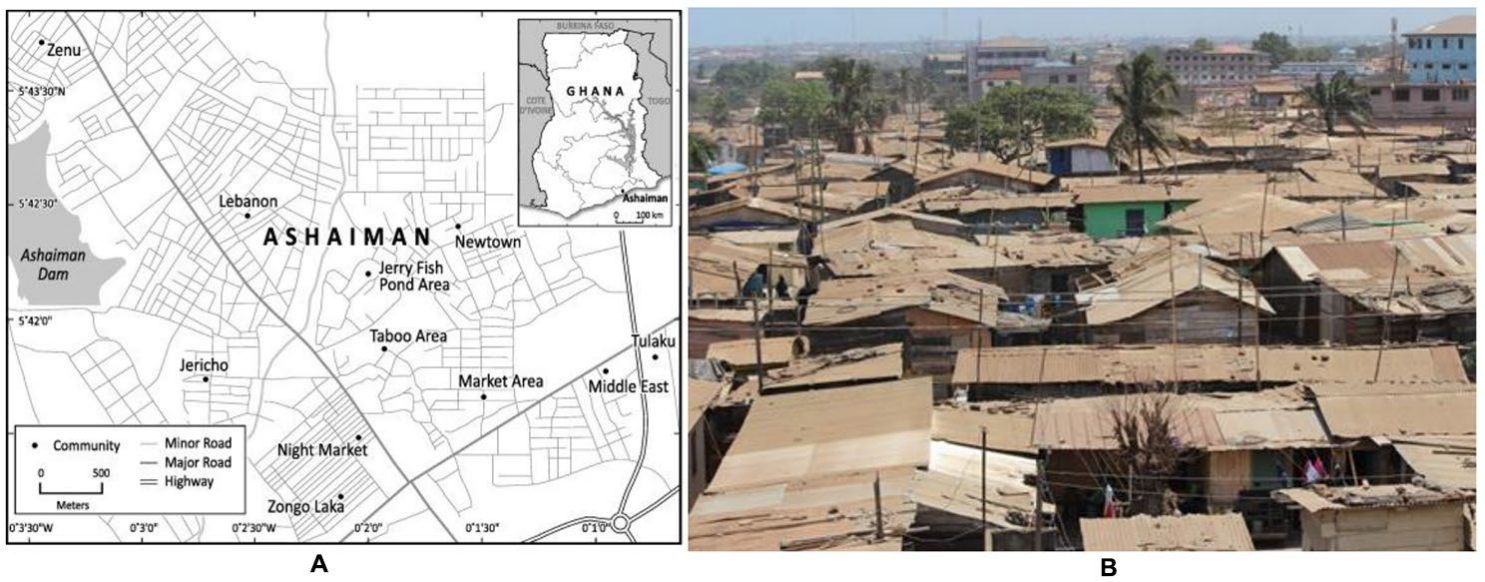
Geographic map (A) and aerial view (B) of Ashaiman-Ghana, participants from Zongo Laka, Night Market and Market Area. Images were taken in 2019 and culled from Google maps in February 2023

According to Dr Richard Bofah, the country coordinator for SDGs, who gave highlights of the National Development Planning Commission (NDPC), Ghana’s report at a regional dissemination workshop, a population of 8.8 million people live in slums. Of the 23 slums in Ghana; Accra, Tema-Ashaiman, Kumasi, Tamale and Takoradi are listed as the predominant urban areas with slums. This study was performed in two slum communities (i.e., Teshie and Ashaiman) in Ghana: a fishing area and an industrial area respectively in the Greater Accra region. Teshie is a settlement with a dominated population of the Ga tribe while Ashaiman is a mixture of tribes from all over Ghana. Houses in Ashaiman slums are made of containers and wood while those in Teshie usually have mud or cement. Older adults in Teshie will normally live alone in a room but, next door are some family members; however, the same population in Ashaiman living alone have their family in the next town or the rural area. The diversity of these two slums influenced the selection choice as we wanted an overall view of the health and social needs. Figures 2 and 3 show the pictorial image of the study setting.

### Participants and recruitment

Older adults were selected based on the following criteria: (1) aged at least 60 years (retirement age in Ghana); (2) lived in one of the two slums for at least 1 year; and (3) gave consent to participate. People who were severely ill, e.g., who had no energy to go through the interview session, were excluded from the study. The first author PYAA asked participants who had previously participated in a study that examined the quality of life of older adults in slums [37] for this study. Next to this, a snowballing technique was used to recruit the remaining participants. People who participated in the interview were asked if they knew others who were potentially interested in participation. This technique was most appropriate because of the nature of the slum arrangement [39]. Additionally, it has been widely used in similar settings [40–42]. If an older adult met the eligibility criteria, the purpose of the study was explained, as well as information on confidentiality. The interview was conducted by the first author who has experience in conducting qualitative interviews. Data collection continued until saturation was reached.

Research Questions.

1. What is the slum-dwelling older adults’ perception of health and social care?
2. What are the health and social care needs of slum-dwelling older adults?
3. How do the health needs of slum-dwelling older adults influence their QoL?
4. How do the social needs of slum-dwelling older adults influence their QoL?
5. How do older adults in slums cope with these potentially unmet needs??

### Study instrument

A semi-structured open-ended interview guide was used to collect data from each participant. The guide was developed based on the research questions which stemmed from two pillars of the WHO policy framework on Active Ageing and in consultation with the literature on health (care) and social needs [34–36, 43, 44]. After developing the first draft of the guide, two qualitative methods experts reviewed the guide and gave suggestions for improvement whereafter the guide was pilot tested among some older adults in another slum with similar characteristics and adjusted. These suggestions were mainly focused on additional probes for the selected topics. Topics in the interview guide included (a) the current health situation/experiences of participants; (b) their health needs; (c) experiences with healthcare personnel at healthcare facilities; (d) their social needs; (e) their influence on the quality of life in old age; and (f) coping strategies adopted to meet their health and social needs. Questions asked were further probed to get more information about participants’ responses. In addition, the background characteristics of participants were gathered, including information on gender, age, place of residence and religion. The interview guide has been added in Appendix 1.

### Data collection procedure

Data were collected between May and June 2021 in the homes of the participants using face-to-face audio-recorded interviews. The first author, and two trained graduate nurses, fluent in the local languages (Twi and Ga) conducted interviews. The purpose of the study was explained orally and subjects who agreed to participate were given an informed consent form to sign or thumbprint. Interviews were done immediately afterwards, and audio recorded. The interviews lasted between 45 min and 1 h.

### Rigour (Trustworthiness)

Analysis was done after the first 3 interviews so that new themes that are seen in the responses can be better probed in subsequent interviews ensuring that emerging themes were better probed in successive interviews. The same interview guide was maintained for all interviews to certify consistency. Detailed field notes were kept which allowed for verification and understanding of responses. Direct verbatim quotes were used to buttress the views of older adults. To ensure confirmability, the audio recording was replayed when reading through transcripts to confirm that the results did not include researcher bias. Additionally, a selected participant who was literate and able to read was contacted with the transcript of her voice recording to ensure what she said had been properly documented. Finally, all recordings, transcriptions, field notes, diaries and literature reviewed were kept on an external drive with the first author at the university only for the audit trail.

### Ethical considerations

Ethical clearance was obtained from the Institutional Review Board of 37 Military Hospital (37MH-IRB IPN 199/2018). Also, permission from the municipal assemblies of the data collection sites was obtained. Additionally, information on voluntary participation and the right to withdraw was shared with all participants. Confidentiality and privacy were guaranteed by keeping the data of participants protected and restricted. No third party had access to the data (as specified above); this was to ensure the confidentiality of participants is protected.

### Data analysis

Analysis was done using the procedure of reflective thematic content analysis where themes were generated both inductively and deductively [45]. Based on Colaizzi’s phenomenological approach seven steps were followed during the analysis [64]: (1) The first author played, listened to the audio recordings, and transcribed them verbatim. The transcripts were read several times to help the author familiarize herself with the data and make meaning of the narrations given; (2) Significant statements were noted and extracted to a separate sheet. (3) meanings were made from the statements by assigning preliminary codes to the data to describe its content. The Atlas Ti 9 software was used to manage the data. Each interview transcript was uploaded into ATLAS Ti 9 software as separate files for coding. PYAA and MI coded transcripts independently by reading individual sentences critically and allocating words or phrases that captured the meanings of the sentences; (4) patterns or themes were searched for in codes across the different interviews. Similar codes were grouped to form subthemes while similar subthemes were regrouped and refined to construct themes. Codes that fit into the two pillars of the framework for Active ageing were grouped; (5) Review of themes was done to be sure it describes appropriately the perceived health and social care needs of the sample population. The field notes served as an additional context for interpretation in making decisions about the codes and themes; To ensure that the data were well represented, a series of meetings were held between (Blinded for review) to build consensus on the agreed themes and subthemes; (6) the complete structure of themes and subthemes was used to produce the report; (7) Finally, validation of the findings was sought from some research participants to compare the researcher’s descriptive results with their experiences. Four main themes and fifteen subthemes emerged from the transcripts (See Table 2). The COnsolidated criteria for REporting Qualitative research (COREQ) were used to guide the reporting of this study [46].

## Results

### Background characteristics of participants

A total of 22 interviews were conducted whereafter saturation was reached. After these 22, three more interviews were performed to conclude that indeed saturation was reached. Overall, 25 participants were interviewed, and fifteen (60%) of them were females. The ages of participants ranged from 60 to 86 years. These are summarized in Table 1a. Details are given in Table 1b in the Supplementary file (Appendix 2).

**Table 1a:** Background Characteristics of participants *from Ghana, taken 2021 (study on the health and social needs of older adults)*

Characteristics of participants	Categories	Frequency(n)	Percentage %
AGE (YEARS)	60–65 66–70 71–75 76–80 > 80	12 8 3 1 1	48 32 12 4 4
GENDER	Male Female	10 15	40 60
RELIGION	Christian Moslem None	15 4 6	60 16 24
EDUCATIONAL LEVEL	Primary Secondary Illiterate	9 7 9	36 28 36
MARITAL STATUS	Single/ Never married Married Widowed Divorced	3 11 8 3	12 44 32 12
SOURCE OF INCOME	Vocation Trading Family Friends	6 7 11 1	24 28 44 4
LIVING ARRANGEMENTS	Spouse only Family Alone	1 17 7	4 68 28

### Organization of themes

After analysis of transcripts, four main themes were identified: older adults’ perception of health, (de)motivators of health service use, challenges of older adults, and coping strategies. Overall, eighteen subthemes were generated from the data. The themes and their corresponding subthemes are presented in Table 2.

**Table 2:** Generated Themes and sub-themes

Themes	Sub-themes
Older adult’s perception of Health	Physical disease conditions Mental well-being Relationships with people Superstition
(De)motivators of health service use	Perceived cause of health condition Healthcare providers attitude Challenges with the National Health Insurance Scheme (NHIS) Effectiveness of medications Length of waiting time Proximity of major health facilities
Challenges of older adults	Isolation Need for assistance with activities of daily living Need for financial assistance for basic care Sleep deprivation Limitation in activities Stigma by health workers
Coping strategies	Social support Engagement in Religious activities

### Older adults’ perception of health

An individual’s perception of health is an important influence on their QoL. This theme described the views of older adults about what health is, their current health status, and their view on factors that influence it. Participants felt that their health referred to their physical health, disabilities, handicaps, having a sound mind and being at peace with people. They mentioned that the ageing process, and “external powers” influence an individual’s health. Some mentioned their current disease conditions either medically or self-diagnosed. Physical disease/medical conditions, mental well-being, relationships with others and superstition were captured as subthemes. These are described below with supporting quotes from participants.

#### Physical disease conditions

Participants (15) mostly reported having chronic diseases such as hypertension and diabetes meant they did not have good health and were taking medications for these diseases.

*“The only challenge I have now is High blood pressure. The last time I went for a check-up was around February… I buy my drugs from the pharmacy here” ***TOA3**

#### Mental well-being

Participants stated that if you can reason and think normally like everyone then you are healthy. The mental well-being issues described in their view were mainly influenced by old age: forgetfulness, and excessive thinking about death. The majority (22) of the participants said they easily forget and require support in finding their misplaced items when they need them for IADLs. This also hinders their autonomy.

*“I easily forget things… with my current state, I just pray for death because I cannot move anywhere always in bed. Is this what growing old is about?*” **AOA3**

A few participants (3), however, said they easily remembered things. One woman specifically mentioned that she was very “smart” in remembering things.

*“…me I don’t forget things … I am very smart…I easily remember things” ***TOA1**.

Participants stated they were usually lost in their thoughts regarding one issue or the other. One participant held grudges from the past with other family members. Other older adults narrated that they think about their age mates and loved ones who had died when alone. They think excessively.

“*My daughter* (referring to the interviewer*), … I can’t help it. I think a lot. To give birth and all your children turn their back on you is not a pleasant experience” ***TOA8**.

#### Relationships with others

Participants also mentioned that being at peace with people meant you are healthy. In describing their social health, all participants stated they could easily form relationships with others. Most participants expressed gratitude to technology with the development of mobile phones (non-sophisticated) as this enables them to communicate with relatives, even if they live far away.

*“They call me, and I also call them, so they don’t feel so far away…thanks to the phone” ***AOA12**.

Eight (8) participants expressed their religion as an important part of their happiness. They maintained that having a close relationship with the church or another religious body, even after being home-bound due to immobility keeps them cheerful.

*“I miss the fellowship with the bigger church. Because of my arthritis, I don’t attend church nowadays ... But they come to give me communion every month” ***AOA11**

Widowed participants expressed they missed the companionship of their deceased spouses. They expressed the emptiness created had greatly affected them. They feel lonely and sad as there is nothing to engage in.

*“Things have not been the same since my husband passed away… with my knee pains also I can’t farm so I rely on my sisters whenever I need something…there’s nothing for me to do. I sometimes feel lonely and sad” ***AOA11**

Some female older adults became caregivers of their spouses and could hardly attend any social function.

*“….my husband is battling with illness and has become bedridden, so I take care of him and the home … I cannot go anywhere … I don’t have money to employ anyone to take care of him for me” ***AOA4**

#### Superstition

Various participants were under the observation that their current health status was a result of “external powers”, and they believed nothing could be changed about their health status. For instance, one participant who experienced a stroke was under the impression that this was the result of a colleague who envied him because he was “the bosses’ favourite”. Another older adult who experienced a stroke perceived he had been bewitched by his spouse.

*“I have had a stroke for about 3 years now. They said my blood pressure was up, but I was not aware…I know it was my colleague from work who did this to me” ***AOA13**

*“I came back from a work trip and my wife had left with the children. Some days after I developed a stroke. I believe my wife had a hand in it… but I leave her to God” ***TOA8**

### (De)motivators of health service usage

Participants expressed that their use of health services was influenced by various factors. Some were individual concerns, beliefs in one medication or the other and various challenges with the health sector. Sub-themes that emerged in this topic were ‘perceived cause of health condition’, ‘healthcare providers’ attitude’, “challenges with the National Health Insurance Scheme (NHIS)”, “effectiveness of medications”, “the length of waiting time at health facilities”, as well as “proximity to major health facilities”.

#### Perceived cause of health conditions

Older adults have varying views on the causes of their diseases. Under this theme, the perception of the health status of older adults contributed to whether they will access healthcare or not. Participants who perceived their current health condition to be because of someone bewitching them did not see it necessary to visit the healthcare facility. They stated that they will not receive a cure for their disease if they go to a hospital, because it is a spiritual battle, not a “science” one.

*“…I don’t go to the hospital also because this was caused by my former wife spiritually so the hospital cannot reverse it…” ***TOA8**

Others who viewed their health condition to be a result of poor lifestyles and changes in the ageing process will visit a health facility for treatment.

*“I see when I’m passing stool that I’m sick. I feel very constipated all the time… My feet also hurt when I walk for a while, old age… I plan to visit the hospital”****TOA4***

#### Healthcare providers attitude

Under this theme, participants stated that the attitude of some healthcare providers influenced older adults’ usage of healthcare services. participants described some professionals as being nice or friendly, others were rude, and some complained that some were selective in who to be nice too.

*“They are sometimes nice. At times also they are busy so when I go to the hospital, they may not notice you …” ***AOA3**

Others felt that health professionals gave preferential treatment to patients they know. Therefore, they rather practice self-medication and not waste their time going to the hospital.

*“They do their work and I also watch them. I don’t have a friend there, so I wait. Usually, those who know the nurses and doctors are moved ahead fast in the queue” ***TOA2**

#### Challenges with the national health insurance scheme (NHIS)

Another important factor influencing health services use is issues with the NHIS. Some issues discussed included expired cards, financial difficulties renewing cards, poor services provided to those with valid cards, non-subscription to the NHIS and limited coverage of services provided by the scheme.

Most participants (20 out of 25) did not have a valid National Health Insurance Scheme (NHIS) card due to financial challenges.

*“I previously used the card but when it expired, I currently don’t have money to renew it…” ***AOA12**

Participants narrated that when they utilised the NHIS card, the standard of services provided was not acceptable. They were also not treated with respect because health professionals felt they were not paying for services.

“*When you go with the card, there is no rush to attend to you, they lump us together at one corner and attend to those who pay out of pocket…” ***AOA9**

Some older adults narrated that they have never had an NHIS card. They explained that queues for patients on the NHIS card are usually long and stagnant, so they preferred to seek healthcare from pharmacy shops.

*“I have never subscribed to the scheme… I prefer to buy from the pharmacy than to go to the hospital… The queues are too long, and the services provided are poor” ***TOA7**.

There were also complaints about NHIS not providing a lot of health services which could have benefitted older adults.

*“The health insurance does not cover my physiotherapy… but most of my drugs are covered” ***AOA3**

#### Effectiveness of medications

Participants had diverse views and beliefs regarding the potency of the medications prescribed.

Some participants felt that conventional medications were not working as expected, and therefore looked for alternative treatments, such as herbal therapies.

*“I want to be able to walk well but still nothing is happening that is why I am doing herbal” ***AOA3**

However, this view was not shared by everyone: some participants preferred conventional medications as they viewed herbal medications as not safe.

*“I have heard of people who have had reactions and even died after taking herbal preparations. I don’t trust herbal medicine…I don’t think it is safe” ***TOA3**.

#### The length of waiting time at health facilities

Almost all participants complained they had to wait a long time to be served at the health facility.

*“…if I have to go to the hospital, I have to get up very early otherwise I join a long queue and spend the whole day there” ***TOA2**

#### Proximity to major health facilities

All participants admitted that there was a health facility close by. However, they explained that there was limited health care provision in these centres. For instance, in the Teshie slum, they only provided first aid. Most participants expressed that larger healthcare facilities were situated far away and therefore, transportation is required.

*“… the clinic at the centre is hardly active. You only meet people who come for weighing children…but for us the old people we need the bigger hospital which is far from us…” ***TOA5**

### Older adults’ interpretation of social care

Participants expressed that being in good relationships, supported by family and friends, having some assistance with laundry, cooking etc., and receiving financial assistance for their medications and day-to-day needs meant that they cared for them. Sub-themes that emerged in this theme are “good relationships with friends and family”, “assistance with Instrumental Activities of Daily Living (IADL)”, and “financial assistance for health and basic care”.

#### Good relationships with friends and family

All participants except one had a good relationship with their family members. Even when not living together, they maintained communication.

*“We are on good terms. We call each other and meet occasionally at funerals and family meetings” ***TOA5**

#### Receiving support with instrumental activities of daily living (IADL)

Some participants (16) were fortunate to have neighbours and family around who assisted with shopping and washing. They appreciated this and said it took a burden off. They could not imagine having to do everything by themselves.

*“…my grandchild here helps me wash my clothes and … I send her on errands so it helps me a lot.” ***AOA8**

#### Having financial support

Participants stated that financial assistance from their children and some family members helped them to meet their day-to-day needs. Some relied solely on their children for purchasing medications. When these are not forthcoming, they become very disturbed. Some also sell drinking water so they can save some money for difficult times.

*“My son always sends money for my medicine. I sell this bottled water here to those who come to the church so that I use the money for myself.” ***TOA2**

### Challenges of older adults

Participants narrated that they have challenges with social care as well as health care and these influence their quality of life. They mentioned needs like companionship, financial assistance for their basic needs and assistance with washing, shopping and sometimes cooking. They also stated they experienced difficulty sleeping, and mobility problems, and sometimes they feel stigmatized by health workers. Sub-themes that emerged are isolation, assistance with activities of daily living, and financial support, sleep deprivation, limitation in activities and stigmatization by health workers.

#### Isolation

Most participants stated that they will love to have people visit them to keep them company. One participant narrated he had been neglected by his wife and children for a long time. Additionally, his extended family members (family members who are not spouses or children) have ignored him as he currently has no money and had problems with his mobility. He feels isolated.

*“……I was here with my wife and the children before she left with the children…I don’t know what she told them, so no one visits me… No one visits me, they only wave at me when going to family meetings…” ***TOA8**

#### Assistance with IADLs

Some participants mentioned they were unable to perform instrumental activities of daily living easily usually because of immobility and needed assistance with various tasks, such as mobilising, washing, doing laundry, and going to the market.

*“I cook food for myself, but I need help, especially with going to the market and washing some clothes… I do get help sometimes” ***TOA3**

*“…I can’t do anything for myself, I need to be carried to the washroom and everywhere…” ***TOA8**.

#### Financial assistance for health and basic care

Irrespective of the age of the participants, many of them were still working to be able to financially support themselves. Still, they argued needing financial assistance as well for basic care.

*“…the sachet water I sell here is not enough to feed and pay medical bills for my husband …if the government will come to our aid, we will be grateful” ***AOA15**

The study also explored the impact of perceived needs on the QoL of slum-dwelling older adults. Most participants said they encountered sleeping difficulty for one reason or the other. Reasons ranged from noise in the environment, and body pains to missing loved ones and worrying about money for upkeep.

*“I will say that I struggle to sleep most often because of the noise from the bar opposite. Also, I have pains all over my body…it makes me very dull and sluggish during the day.” ***AOA6***“… I am always thinking about my late husband and my friends who have passed on…my husband was my companion and source of financial support. My life has not been the same since he left last year.” ***TOA9**

A few slum-dwelling older adults explained that mobility problems limited their participation in activities they would have wished to engage in. Others also mentioned that the absence of meeting places for older adults restricted the social activities of older adults in the slums.

*“I am not able to move about on my own because of the stroke. I used to attend family gatherings but not anymore…and no one visits me” ***TOA8***“aside from this big tree there is no meeting place where we can sit and socialize as older adults. We have to always be indoors or sit in front of our house. When it rains, we can’t sit under the tree…even the front of this house gets flooded*(laughs).*” ***AOA7**

Some participants were deterred from visiting health facilities because of perceived stigma and searching for alternate treatment when they are not well.

*“…they* (health workers) *are selective in who they attend to first. I join long queues and stay for hours in the hospital…I look for other treatment when I am not well.” ***AOA13**

### Coping strategies

The theme of coping strategies referred to how older adults managed their socioeconomic and health needs. Participants either supported themselves by engaging in petty trading, or other menial jobs, or relying on others for support. Two subthemes emerged: social support and religious engagements.

#### Social support

Physical support, financial support, and self-support were themes that emerged from the subtheme ‘social support’. Participants mentioned that they received some physical and financial support from family members and friends most of the time. They expressed that because of their ability to develop relationships, they could also rely on people who are not family members to assist with things in the home. However, they also do a few things to support themselves.

#### Physical support

Participants discussed that they sometimes received support for healthcare, and IADL from their children, good Samaritans, and neighbours. Additionally, extended family members provided updates on family meetings for participants with mobility problems.

*“…good Samaritans sometimes pass by to visit but I wash my clothes by myself, and my sisters also help” ***TOA4**

*“I am glad my wife is around me because she helps me greatly and I owe it all to her support” ***TOA10**

#### Financial support

Most participants were not previously formally employed and therefore do not receive pension remittances. Children of older adults were the main supporters of their parent’s finances. More than half of the participants reported that their children provided financial support for either food or medications.

*“My older children usually send me something (*money) *every month for our upkeep. I am not working now because at 71 I am very old” ***AOA13**

Siblings also gave support for food. Other family members also provide some financial support. Those who lived alone received donations from some family members who visit.

*“I help my sisters to prepare kenkey* (food made from ground corn) *for sale and they give me food when we are done” ***TOA4**

*“I don’t work because of my age, and I am not strong enough. … other family people who visit me give me money” ***TOA7**

Sometimes neighbours also help. Given the uncoordinated arrangements in the slums, the entrance to someone’s house is someone’s place for selling. These neighbourly sellers also provided financial support for some older participants in the slum.

*“… my child supports me, but she also goes to work so this lady here selling charcoal comes to my aid and sometimes buys food for me” ***TOA8**

#### Self-support

To help meet their financial needs and be engaged, most participants were involved in some form of activity for money for their daily upkeep. Older adults stated that they did not want to burden their children and were reluctant to depend solely on their children for financial and social support.

*“My children are working and have the means to help but I decide not to be a liability. Even though they will help when I ask, I am also selling. It also keeps me active” ***TOA3**

*“I can wash my clothes and cook my food. I buy my ingredients from a woman next door, so I don’t need to go to the market. I don’t like disturbing people, so I try to do things by myself” ***AOA11**

Some older adults who live alone explained that they help themselves by working and doing what they can to get money as they often buy food from vendors.

*“…I live alone, and this place is not big, so I do everything myself…because I am a driver, I buy food from the station. I only come home to sleep….” ***AOA14**.

#### Religious engagements

Participants relied on religious engagements to relieve sadness, loneliness, and boredom. Religiosity is one major aspect of the life of Ghanaians and most especially older adults.

Some older adults expressed that talking to God minimizes sadness.

*“I get sad when I hear my age mates are dying. When I am sad, I chat with God. I pray and discuss a lot of issues with Him” ***TOA4**

Other participants sang hymns to relieve idleness and loneliness.

*“I was a chorister, so I sing hymns when I am alone” ***AOA8**.

A few older adults explained that reading the Bible and preaching to customers were some religious engagements they employed to cope with boredom.

*“If I am to be idle, I engage myself in reading the Bible” ***TOA1**.

## Discussion

This study aimed at exploring the perceived health and social care needs of older adults in two slums. When comparing responses from the two slums on their views on health and social care needs, they all have similar views. The main variation was in their “financial needs”. Most people in the slum close to the industrial area were actively engaged in informal jobs and could have enough money to care for themselves. Almost all the participants who lived alone were also in this slum. So, there is a high probability that they had a low dependency rate therefore they could manage their finances and did not have to rely so much on others.

Perceived health needs were mainly current disease conditions (arthritis, diabetes, hypertension, vision/hearing challenges), challenges with health insurance, the behaviour of some health professionals, the proximity of health facilities, and unnecessary queues at major health facilities. Unmet social needs identified by this study were a sense of neglect by family (need for companionship), requiring assistance with activities of daily living, and the need for financial support. Older adults often had difficulty sleeping as they thought of how to meet their needs and also waited on death. Generally, it was observed that there were more perceived health needs than social needs among these older adults. This leads to the question of whether older slum-dwellers perceive health-related needs as more important.

Our study found nine perceived issues related to the health of older adults living in slums: (1) disease condition, (2) mental well-being, (3) relationships with others, (4) attitudes of health care providers, (5) National Health Insurance Scheme (NHIS), (6) effectiveness of medications, (7) proximity to health facilities, (8) superstition and (9) length of waiting time in health care facilities. The themes fit into the two pillars of the WHO policy framework on Active Ageing which was used in the study conceptualisation.

To improve self-perceived healthcare needs, participants mentioned that access to healthcare facilities should be improved by (a) having more well-equipped health facilities close to slums, (b) reducing waiting time for healthcare services, (c) decreasing costs for healthcare use by restructuring the NHIS, (d) increasing and improving the services provided under the insurance scheme. Furthermore, how an individual perceived his/her health condition also influenced the use of modern health services. An example is that participants who attributed their illness to spiritual powers such as “bewitchment” are not likely to visit modern healthcare facilities for treatment. A reason for this is that in African countries, spirituality is often regarded as an explanation for many occurrences. These findings are consistent with studies in Tanzania [47] and Malawi [48] where bewitchment and spirituality were linked to eclampsia and anaemia. The belief that spirituality influences health status, made participants believe that medications were unable to reverse their health status, influencing healthcare use. Therefore, educating slum dwellers on finding, understanding, appraising, and applying health information to make health-related decisions, also known as health literacy, might improve their self-perceived health [49]. Health education should take the strong spiritual and religious beliefs of this population into account.

In the slums, as most older adults are low-income earners compared to formal settlement, financial constraint is a major barrier to the utilization of healthcare services among older adults living in slums. This finding is consistent with that of Fayehun, et al. [50], and Cadmus, et al., [51] which were conducted in Nigeria. The findings on the use of the NHIS card, the attitude of health professionals, and the length of waiting time influencing the healthcare use of older adults also agree with findings by Agyemang-Duah, et al. [52] in a rural Ghanaian community. The NHIS is supposed to be free for all older adults above 70 years however this does not seem to be the case. Participants also complained about the poor and inadequate services provided under the NHIS. As most older adults in this study had expired health insurance cards, they often purchase medications from the pharmacy or prepare herbal medications when not well. This confirms a study by Awoke et al., [53] and Amiresmaili, et al., [54] which postulated that possession of health insurance cards influenced the utilization of healthcare services.

In this study, we also observed that some participants patronized pharmacies and herbal preparations more than the hospitals. The findings also confirm a study in Mumbai by Naydenova, et al. [55] where some participants utilised pharmacies and alternative medications instead of the healthcare facility.

Older adults in this study perceived social care to be having good relationships with family and friends and receiving both physical and fiscal assistance for basic care needs. This is similar to previous studies where participants referred to social care as having support from family and friends, support for self-care and instrumental support (monetary) [60–62]. It appears that the unmet social needs among older slum-dwellers were fewer than expected based on the living conditions in the slum. Most participants appreciated social support from family and friends. This finding confirms the quantitative study by Attafuah et al. [37] where the older adults in the slum appeared to have a moderate QoL in the social domain. Findings from this study revealed three social needs (1) companionship; (2) assistance with IADLs; and (3) financial assistance for food and medications. When looking at companionship, most participants desired the company of friends and family, but they had to make do with phone calls because of distance. One participant felt he had been neglected by family and close friends. When looking at the other factors influencing social needs, most participants argued that they needed assistance with IADL and financial support for basic needs. These were also found by Iriarte, & Jimenez, [56] among ethnic/racial minority groups in Chile. Married men in the current study viewed their wives as their main support system, consistent with findings by Tkatch et al., [57]. According to Iriarte, & Jimenez [56], a caregiver must be healthy to care. However, in our study, some older adults who are not physically fit themselves were caregivers of their spouses because there is no one to take up the role. In agreement with Cash, et al. [58], caregiver responsibility is seen as an expectation in marriage. This perspective additionally supports research on both the benefits of social support and the reciprocity of social support exchanges or being able to both give and receive, as having significant benefits for older adults in slums. According to Akinrolie, et al., [59], the feeling of reciprocity could be the reason why children were the main social support system for older adults. This also affirms the bond between children and parents in the Ghanaian setting despite the breakdown of the extended family system. Another finding was that most participants were currently engaged in menial jobs because as they stated, they did not want to depend too much on their children. This occurs as most slum dwellers are into non-formal employment and hence do not benefit from pensions in their old age. Also because of the high level of illiteracy in the slum, private pension schemes are not widely known.

On the influence of the perceived health and social needs on their QoL, participants mentioned that they had difficulty sleeping because of pain in their joints. Additionally, they have mobility issues, and this restricts participation in activities. At the health facilities, they sometimes feel stigmatized by health workers coupled with feelings of loneliness from family neglect/absence hence they try to keep to themselves. This prevents them from going to health facilities and negatively affects their QoL. Generally, having a good perception of health and social care issues has a positive influence on QoL as stipulated by Ingrand, [63]. Participants who harboured superstitious beliefs about disease conditions do not rate their QoL as good and had issues with everyone around them.

This study also sheds some light on strategies older adults used to cope with unmet needs. It is observed that slum dwellers are better able to cope with unmet social needs than with unmet healthcare needs. Even though some participants expressed a feeling of neglect from family and friends, most older adults were satisfied with the family support received. Firstly, most participants narrated they received some form of support from family and friends to help cope with their health and social needs. Children in the African setting are expected to be the primary social support for their parents. Therefore, for more than half of the participants, children provided financial support for either food or medications for older adults in the slums. Secondly, to cope with unmet social needs, older adults engaged in religious activities such as singing and evangelising to people to form relationships. Religiosity is very prominent in the African setting, and this is therefore not surprising especially in the slums as most participants showed over-reliance on God with hopes for survival and getting a better quality of life. Health education in churches could be emphasized to improve the health and social needs of the populace. Lastly, participants said they supported themselves as much as possible either by engaging in a trade or cooking their meals. This could be attributed to the need to be active and maintain autonomy as they aim to be less dependent on others.

Understanding the perceived health and social needs of older adults living in slums can help health workers in providing appropriate care. The uneven walkways and distance to major health facilities for example can be temporarily managed if health workers especially community health nurses are committed to rendering services at the doorsteps of older adults in the slum. Additionally, policy development can be directed towards providing geriatric services close to slum neighbourhoods.

## Strengths and limitations

To our knowledge, this is the first study exploring the health and social needs from the slum-dwelling older adult’s perspective in Africa. Because of the qualitative nature of the study, participants had the opportunity to express themselves freely in their local languages. Varieties in background characteristics between participants were sought to increase the internal generalizability of research results in the slum setting. However, external generalizability may be difficult as additional studies in comparable contexts may reveal new meanings. A limitation of this study is that there was a lack of privacy when performing the interviews due to the slum setup: other people from the slum setting were often present and listened along. This could have influenced the answers given by participants. Secondly, older adults who were interested in participating received a breakfast package after the interview. This could have also led to selection bias. Nevertheless, the participants in this study provided significant insight into a general perspective of health and social needs that can provide researchers and clinicians with knowledge of what older adults in slums may need to improve and sustain their health. Future research should consider expanding these insights through larger populations of more slums.

## Implications

This study underscores the need for improved access to health and social care services for older adults living in slums. Policymakers are advised based on the results of this study, to consider restructuring the NHIS regarding price and services provided under the scheme for older adults. In addition, the provision of well-equipped, older adult-friendly health facilities close to slums will decrease the issues of proximity and waiting time. Religious leaders should be involved in promoting health education activities among their congregations.

## Conclusion

Participants discussed more healthcare issues than their social care needs. Health-related issues included their understanding of their health status, health insurance challenges and the attitude of health professionals. Social care needs largely emphasized by most participants related to companionship. This study presents an important understanding of the health and social needs from the perspective of older adults in the slums as this affects their overall quality of life. The provision of formal services such as improved home visits by healthcare professionals can assist in the individual education of older adults on their health needs and how to manage them. Lastly, older adults receive some social support from family and friends, but this is not consistent. Hence the creation of daycare centres for slum-dwelling older adults in nearby communities will enable socialization with peers.

## Electronic Supplementary Material

Below is the link to the electronic supplementary material.

Supplementary Material 1

Supplementary Material 2

## Data Availability

Data is available from the corresponding author upon reasonable request.
