# Supplementary material for: Health and social needs of older adults in slum communities in Ghana: a phenomenological approach used in 2021
Source: Arch Public Health. 2023 Apr 27;81:74. doi: 10.1186/s13690-023-01056-9 (PMC10134515; doi:10.1186/s13690-023-01056-9)
Supplement: Supplementary file 1 — Supplementary Material 1 [file 13690_2023_1056_MOESM1_ESM.pdf]

Title of study: Health and social needs of older adults in slum communities in Ghana: A phenomenological approach

INTERVIEW GUIDE

Date of Interview: ___________________________________

Start: ____________ End:____________________

Pseudonym____

Section A

DEMOGRAPHICS

AGE

Gender: Male ☐ Female ☐

Ethnic group:

Marital status: ……………………………………

Educational level: ……………………………………………

Place of residence: ………………………………………………..

Previous settlement ………………………………………………….

How long have you been living in your current settlement?...

How many dependents do you have? ……………………………….

Who do you live with?..

Who can you rely on for support if you need any?..

SECTION B

Current health status and health needs of the older adult

I. Please tell me how is your health currently.

   Probes:

   Physical (movement: eg. walk with or without aid, body aches? Other activities? Exercise? etc)

   Social (work? Church? Community group? Family meetings or visits? Vacations? etc)

Psychological (feelings, emotions, do you easily remember or forget things etc)

Chronic conditions (any medical diagnosis? Family disease? Minor undiagnosed ailments?)

Current medications (prescribed or self-medication?)

Ability to take medications (like or dislike, forgetfulness, reactions Chronic conditions

Side effects/problems with medication

Other therapies?

Health-seeking behaviour and treatment pattern.

Health insurance?

II. Please tell me how you manage your health

Probes:

Easy access to medical care?

Health facility (Government hospital, Private hospital)/ Traditionalist/ Pharmacy shop

Traditional/ herbal medication

Experiences with healthcare services (nurses, doctors, OPD?)

Lack of availability, Distance, No health insurance, Financial difficulty, Attitude of staff, Long queues/waiting time too long

Are you satisfied with access and medical service?

Describe what you expect in a perfect health care system or looking at your current situation, when is health care adequate in your view?

Social needs and challenges

III. Can you tell me how you relate to friends and family?

Probes:

Isolation (what is your normal day like?)

Boredom (how often do you find yourself sitting alone and doing nothing? How about sleeping in bed? What are the usual things you do and are not happy doing?)

Family (communication pattern, frequency)

Social support (community services and engagements)

Financial support (pensions? any remittances? donations, insurance?)

Religious activities

Friends/Neighbours

Family activities/Weddings/birthdays/funerals/ outdooring [naming ceremonies] (frequency, roles played)

What are some challenges you face?

How is this affecting your quality of life?

Coping

IV. Kindly share with me how you have been coping with life as an older person.

Probes:

Family (spouse, children, grandchildren, other family relations, non-blood related household members eg co-tenants, house helps etc.)

Friends/Neighbours (old schoolmates, former work colleagues, former girl/boyfriends, church groups etc.)

Financial support (active working? pension? Gifts, begging for alms?)

Physical support (walking aids?)

House chores (self-service or assisted?)

Market

Food (eating pattern)/ cooking food choice, food tolerance, and changes?

Would you prefer to always live here?

V. Is there anything else you would want to share?

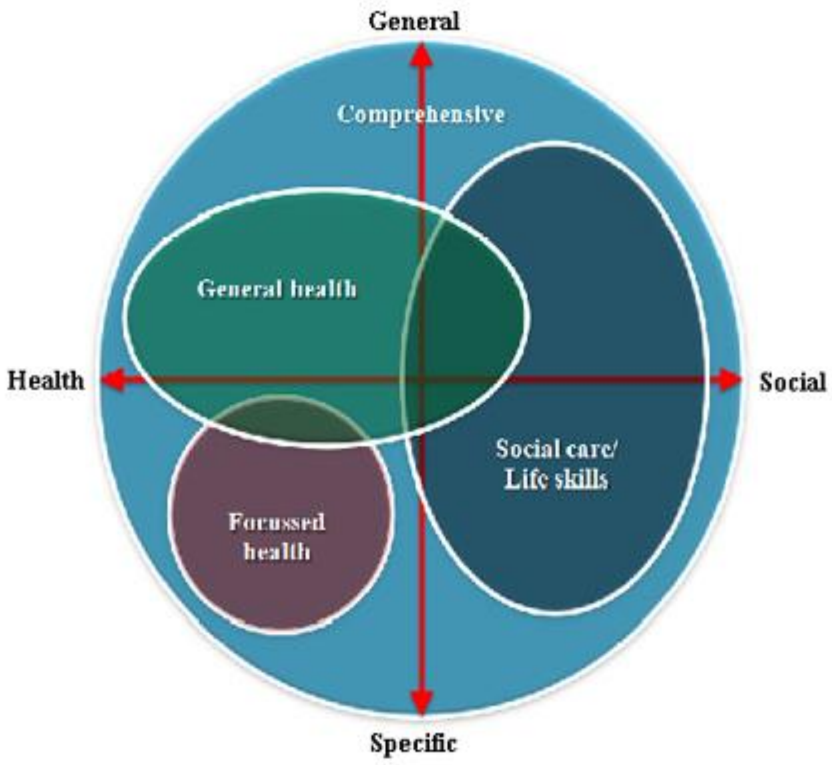

Graphic 1. Types of self-perception with the different dimensions and interactions

Taken and adapted from: Griffiths P, Ullman R, Harris R. *Self-assessment of health and social care needs by older people: a multi-method systematic review of practices, accuracy, effectiveness and experience*. London: NCCSDO, 2005. [Date

- ***Health care approach.*** This domain focuses on assessing specific health aspects and problems. To identify older adults with hearing and vision loss, and problems with nutrition, mobility, and function. This type of self-evaluation can help to predict current needs and some future ones.

- ***General health care approach.*** This domain is used for evaluating a broad range of factors related to health care. The objective here is to improve general health care and mediate in the patients' relationship with healthcare professionals. Mental health status, functional capacity, social contacts, and use of health services are among the aspects investigated in this domain.

- ***Social care and Lifeskills approach.*** This domain focuses on assessing everyday situations like the individual’s ability to meet his/her basic care needs. Support from family and friends are also examined.

- ***Multidimensional approach.*** This domain looks at the approaches the individual uses to cope or manage their health and social care needs
