# Supplementary material for: Health and social needs of older adults in slum communities in Ghana: a phenomenological approach used in 2021
Source: Arch Public Health. 2023 Apr 27;81:74. doi: 10.1186/s13690-023-01056-9 (PMC10134515; doi:10.1186/s13690-023-01056-9)
Supplement: Supplementary file 2 — Supplementary Material 2 [file 13690_2023_1056_MOESM2_ESM.docx]

APPENDIX 2 Table 1b: Detailed demographics of participants

|  | | | | | | | |
| --- | --- | --- | --- | --- | --- | --- | --- |
| PARTICIPANTS ID | AGE  (YEARS) | GENDER | RELIGION | EDUCATIONAL LEVEL | MARITAL STATUS | SOURCE OF INCOME | LIVING ARRANGEMENTS |
| TOA1 | 67 | F | Christian | Secondary | Widow | Petty trader | Children |
| TOA2 | 68 | F | Christian | Primary | Married | Son and petty trading | Son and daughter-in-law |
| TOA3 | 67 | F | Christian | Secondary | Married | Petty trading | Husband and children |
| TOA4 | 69 | F | None | Illiterate | Widow | From sisters | Sisters |
| TOA5 | 61 | M | None | Primary | Single/ Never married | Masonry | With cousins |
| TOA6 | 73 | F | Christian | Illiterate | Widow | Petty trading | Alone |
| TOA7 | 86 | F | None | Illiterate | Divorced | Children | Child |
| TOA8 | 80 | M | Christian | Secondary | Divorced | Child and good Samaritans | Child |
| TOA9 | 72 | F | Christian | Primary | Widow | Petty trading | Alone |
| TOA10 | 65 | M | None | Secondary | Married | Fishing | Wife and child |
| AOA1 | 60 | F | Christian | Secondary | Married | Trader | Husband and children |
| AOA2 | 64 | F | Muslim | Illiterate | Widow | Children and trading | Children |
| AOA3 | 68 | M | None | Primary | Married | From wife and children | Wife and child |
| AOA4 | 62 | F | Christian | Illiterate | Divorced | Trading and children | Alone |
| AOA5 | 65 | F | Moslem | Illiterate | Married | Husband and children | Husband and children |
| AOA6 | 61 | M | None | Primary | Single | Carpentry | Alone |
| AOA7 | 68 | M | Christian | Secondary | Widower | Driver | Alone |
| AOA8 | 70 | F | Christian | Illiterate | Widow | Petty trading/children | With grandchild |
| AOA9 | 63 | F | Christian | Primary | Married | Petty trading | Husband and child |
| AOA10 | 61 | M | Christian | Primary | Married | Driver | Wife, children, and grandchildren |
| AOA11 | 67 | F | Christian | Illiterate | Widow | Petty trading/children | alone |
| AOA12 | 64 | M | Moslem | Secondary | Married | Driver | Wife and child |
| AOA13 | 71 | M | Moslem | Primary | Married | Children | Wife and child |
| AOA14 | 60 | M | Christian | Primary | Single | Driver | Alone |
| AOA15 | 65 | F | Christian | Illiterate | Married | Petty trading | Husband |
